# Changing prevalence and treatment of depression among older people over two decades

**DOI:** 10.1192/bjp.2019.193

**Published:** 2019-10-07

**Authors:** Antony Arthur, George M. Savva, Linda E. Barnes, Ayda Borjian-Boroojeny, Tom Dening, Carol Jagger, Fiona E. Matthews, Louise Robinson, Carol Brayne, the Cognitive Function and Ageing Studies Collaboration

**Affiliations:** 1 Professor of Nursing Science, School of Health Sciences, University of East Anglia, UK; 2 Statistician, Core Science Resources, Quadram Institute Biosciences, UK; 3 CFAS National Co-ordinator, Department of Public Health and Primary Care, University of Cambridge, UK; 4 Medical Student, Norwich Medical School, University of East Anglia, UK; 5 Professor of Dementia Research, School of Medicine, University of Nottingham, UK; 6 Professor of Epidemiology and Ageing, Institute for Health and Society, Newcastle University, UK; 7 Professor of Epidemiology, Newcastle University; and University of Cambridge, UK; 8 Professor of Primary Care and Ageing, Institute for Health and Society, Newcastle University, UK; 9 Professor of Public Health Medicine, Department of Public Health and Primary Care, University of Cambridge, UK; 10 see Acknowledgements

**Keywords:** Depressive disorders, antidepressants, epidemiology, older people, primary care

## Abstract

**Background:**

Depression is a leading cause of disability, with older people particularly susceptible to poor outcomes.

**Aims:**

To investigate whether the prevalence of depression and antidepressant use have changed across two decades in older people.

**Method:**

The Cognitive Function and Ageing Studies (CFAS I and CFAS II) are two English population-based cohort studies of older people aged ≥65 years, with baseline measurements for each cohort conducted two decades apart (between 1990 and 1993 and between 2008 and 2011). Depression was assessed by the Geriatric Mental State examination and diagnosed with the Automated Geriatric Examination for Computer-Assisted Taxonomy algorithm.

**Results:**

In CFAS I, 7635 people aged ≥65 years were interviewed, of whom 1457 were diagnostically assessed. In CFAS II, 7762 people were interviewed and diagnostically assessed. Age-standardised depression prevalence in CFAS II was 6.8% (95% CI 6.3–7.5%), representing a non-significant decline from CFAS I (risk ratio 0.82, 95% CI 0.64–1.07, *P* = 0.14). At the time of CFAS II, 10.7% of the population (95% CI 10.0–11.5%) were taking antidepressant medication, more than twice that of CFAS I (risk ratio 2.79, 95% CI 1.96–3.97, *P* < 0.0001). Among care home residents, depression prevalence was unchanged, but the use of antidepressants increased from 7.4% (95% CI 3.8–13.8%) to 29.2% (95% CI 22.6–36.7%).

**Conclusions:**

A substantial increase in the proportion of the population reporting taking antidepressant medication is seen across two decades for people aged ≥65 years. However there was no evidence for a change in age-specific prevalence of depression.

Depression is a leading cause of disability worldwide and a key contributor to the global burden of disease for all ages.^1^ Older people may be less likely to report symptoms of depression and the presence of comorbid conditions may make depression more difficult to diagnose.^2^ Estimates of prevalence of major depression in those aged ≥75 years range from 4.6 to 9.3%,^3^ but sampling and measurement differences in these population-based studies make it difficult to determine whether these differences are real or artefactual. Evidence from studies across all age groups suggest that prevalence of major depressive disorder declines in later life,^4,5^ although the reverse may be the case for the presence of clinically significant depressive symptoms.^6^ Since the introduction of selective serotonin reuptake inhibitors in the late 1980s, there has been an increase in antidepressant prescriptions issued.^7^ Prescriptions dispensed for antidepressants increased more than threefold in England between 1991 and 2009,^8^ reflecting a trend observed in other Western countries.^9,10^ Establishing whether there are temporal changes in prevalence of depression among older people is a major challenge, requiring large studies undertaken at two or more points with sufficient time lapse, using the same sampling methods, geographical areas, interviewing approaches and diagnostic criteria. Against a backdrop of greater life expectancy and improved health in later life, the Cognitive Function and Ageing Studies (CFAS I and CFAS II (http://www.cfas.ac.uk)) provide a unique opportunity to test whether the prevalence of depression in England among people aged ≥65 years has changed over two decades, between 1991 and 2011.

## Method

The CFAS I and CFAS II are population-based cohort studies designed to assess the changing health of older people across generations. The original Medical Research Council CFAS (CFAS I) included six geographical areas in England and Wales, three of which were continued into CFAS II: Cambridgeshire, Newcastle and Nottingham. Baseline interviews for CFAS I and CFAS II were conducted between 1990 and 1993 and between 2008 and 2011, respectively.

The study design, methods and interview schedule of the two cohort studies were identical, with the exception of a two-stage sampling process used in CFAS I and a one-stage sampling used in CFAS II. In CFAS I, individuals underwent a screening interview (first stage) and then a subset of the screening sample were invited to take part in a further assessment interview (second stage). In CFAS II, screening and assessment were undertaken simultaneously during one interview for the entire sample.

Eligible participants at each centre were those aged ≥65 years and registered with a general practice within the boundaries of the geographical area. Those living in care homes as well as those living independently in their own homes were included. Participants were sampled from lists from the UK system for primary care registration. In both CFAS I and CFAS II, stratified random sampling was used to secure 2500 participants in each geographical area, with equal proportions aged 65–74 years and aged ≥75 years. Participants were initially approached via a letter from their registered general practice. This was followed by a visit from a study interviewer, who undertook the interview if the individual provided written informed consent. For individuals considered not to have mental capacity, as defined by the UK Mental Capacity Act, a request was made to interview an informant, typically a close relative.

The interviews were conducted face to face, by a trained study interviewer in the usual place of residence of the participant. The interviewer captured participant responses on a laptop computer. In CFAS I, baseline (screening) interviews contained questions about sociodemographic characteristics, perceived health, activities of daily living and use of health and social care services. Participants (and/or their informant) were asked about all medications they were currently being prescribed by their doctor as well as over-the-counter medications and supplements. Drug name, dose and frequency were recorded for all medications. At the assessment interview the Geriatric Mental State examination was undertaken. This is a standardised interview for ascertainment of the presence or absence of mental health disorders in older age. In CFAS II, one interview was conducted, which contained both the screening and assessment components. Further details of the approach and interview content have been previously published.^11,12^

CFAS I and CFAS II used the same Automated Geriatric Examination for Computer-Assisted Taxonomy algorithmic (AGECAT) approach^13^ to diagnose dementia, depression and other mental health disorders among participants in the two cohort studies. The presence or absence of depressive symptoms are used to categorise individuals into six levels of depression severity, which can then be collapsed into three groups: no symptoms (level d0), subclinical depression (levels d1 and d2) and case-level depression (levels d3–d5). Subclinical depression is characterised by minor mood symptoms and some non-specific symptoms (e.g. loss of energy, interest or enjoyment). Case-level depression comprises neurotic and psychotic subtypes, with attempts of suicide taking the diagnostic level to d4 or above. For those with more than one diagnosis, the AGECAT algorithm determines a primary diagnosis. Analysis presented here is restricted to those with a primary diagnosis of case-level depression (neurotic or psychotic). Patients were considered to be receiving antidepressants if they reported use of medications categorised within the British National Formulary (antidepressant sections 4.3.1–4.3.4).^14^

### Analysis

All analyses were undertaken using weights, to adjust for (i) oversampling of those aged ≥75 years and (ii) non-response using age, gender and deprivation status based on Townsend deprivation scores^15^ linked to postcode (CFAS I and II). Weights also adjusted for selection for assessment interview (CFAS I only). To account for changes in population structures, prevalence estimates from both CFAS I and CFAS II were calculated by standardising to the 2011 UK population age and gender distribution.

To investigate whether sociodemographic factors (age, gender, care home residence, centre, Townsend deprivation quartile) were associated with study diagnosis of case-level depression or being prescribed antidepressant medication, we used binomial regression models with a log link for each cohort to estimate risk ratios adjusted for each of the other sociodemographic factors. To test for a cohort effect, we used the same covariates for both cohorts combined. We additionally included interaction terms between each sociodemographic variable and cohort to estimate whether the relationship between sociodemographics and depression diagnosis or antidepressant had changed between the two cohorts.

### Ethics approval

The authors assert that all procedures contributing to this work comply with the ethical standards of the relevant national and institutional committees on human experimentation and with the Helsinki Declaration of 1975, as revised in 2008. All procedures involving human subjects/patients were approved by local and multi-centre ethics committees (CFAS I: MREC99/5/22. 05/MRE05/37; CFAS II: 07/MRE05/48).

### Data availability

The data sets analysed during this study are available upon reasonable request from the CFAS team (http://www.cfas.ac.uk/cfas-i/data/).

## Results

In CFAS I, 9345 individuals were eligible and approached to take part in the baseline screening interview in Cambridgeshire, Newcastle and Nottingham, of whom 7635 participated (response 81.7%) and 1457 undertook the assessment interview. In CFAS II, of the 14 228 individuals eligible to take part and approached, 7762 (54.6%) were interviewed. Details of the change in response between the two cohorts have been previously reported.^11^ Supplementary Table 1 (available at https://doi.org/10.1192/bjp.2019.193) describes the samples from each cohort and the numbers included in analyses.

The estimated prevalence of depression among people aged ≥65 years in CFAS I was 7.9% (95% CI 6.3–9.9%) and 6.8% (95% CI 6.3–7.5%) in CFAS II (Table 1), a non-significant decline of around 20% in prevalence in the intervening two decades (adjusted risk ratio CFAS II versus CFAS I 0.82, 95% CI 0.64–1.07, *P* = 0.14). The prevalence of depression was higher among women than men at both time points. There was no evidence of changes in the pattern of prevalence across age groups. The proportion of people aged ≥65 years living in care homes has declined over the period between the two studies,^12^ but prevalence of depression in care home settings was unchanged, with approximately one in ten residents having case-level depression (CFAS I: 10.0%, 95% CI 6.1–16.1%; CFAS II: 9.8%, 95% CI 5.9–15.8%).

**Table 1:** Number with known depression status, depression prevalence and antidepressant treatment, by age, gender and residential status

	CFAS I			CFAS II		
	*N*^a^	Depression, % (95% CI)	Antidepressant medication, % (95% CI)	*N*	Depression, % (95% CI)	Antidepressant medication, % (95% CI)
All	1457	8.1 (6.4–10.1)	4.0 (2.8–5.6)	7723	6.7 (6.1–7.3)	10.7 (10.0–11.4)
Men	531	6.3 (4.2–9.4)	2.9 (1.5–5.4)	3525	4.5 (3.9–5.3)	6.6 (5.8–7.5)
Women	926	9.1 (6.9–12.0)	4.6 (3.0–6.9)	4198	8.3 (7.5–9.2)	13.9 (12.8–15.1)
65–74 years	630	7.1 (5.2–9.7)	4.5 (2.8–7.2)	3812	6.6 (5.8–7.4)	9.8 (8.9–10.9)
75–84 years	554	8.6 (5.9–12.4)	3.7 (2.0–6.7)	2900	7.0 (6.1–8.0)	11.4 (10.2–12.6)
≥85 years	273	9.5 (5.2–16.7)	3.0 (1.5–5.9)	1011	6.1 (4.7–7.8)	11.6 (9.7–13.8)
Men in community						
65–74 years	274	4.8 (2.8–8.2)	2.8 (1.2–6.6)	1859	4.3 (3.5–5.4)	6.2 (5.1–7.4)
75–84 years	166	8.5 (4.1–16.7)	3.2 (1.0–9.2)	1275	4.5 (3.4–5.8)	6.1 (4.9–7.5)
≥85 years	40	4.6 (1.3–15.3)	0	337	4.9 (3.1–7.8)	7.9 (5.3–11.5)
Overall	480	6.1 (3.9–9.4)	2.8 (1.4–5.4)	3471	4.5 (3.8–5.2)	6.3 (5.5–7.2)
Men in care homes						
65–74 years	17	4.4 (0.6–27.1)	5.8 (0.7–33.3)	11	18.8 (4.4–54.1)	3.9 (0.5–25.4)
75–84 years	14	15.7 (3.8–47.0)	5.2 (0.6–33.9)	24	7.2 (1.0–38.3)	34.0 (16.5–57.2)
≥85 years	18	13.4 (3.0–43.8)	9.0 (1.2–44.8)	19	0	19.3 (6.6–44.7)
Overall	49	11.9 (4.8–26.6)	6.6 (1.9–20.5)	54	7.2 (2.2–21.0)	21.3 (11.9–35.1)
Women in community						
65–74 years	329	9.0 (6.0–13.4)	5.9 (3.2–10.5)	1935	8.6 (7.4–10.0)	13.0 (11.5–14.6)
75–84 years	318	8.5 (5.2–13.5)	3.6 (1.6–8.1)	1563	8.5 (7.2–10.0)	14.4 (12.7–16.3)
≥85 years	142	11.9 (5.5–23.7)	2.6 (1.0–6.4)	562	6.6 (4.8–9.1)	10.5 (8.3–13.3)
Overall	789	9.1 (6.8–12.2)	4.4 (2.8–6.9)	4060	8.2 (7.4–9.1)	13.1 (12.1–14.2)
Women in care homes						
65–74 years	9	39.8 (13.7–73.3)	10.3 (1.4–48.6)	7	0	69.3 (29.2–92.5)
75–84 years	55	10.2 (3.5–26.5)	9.6 (3.2–25.7)	38	21.0 (9.8–39.6)	38.6 (23.8–55.9)
≥85 years	70	6.0 (2.4–14.4)	6.1 (1.8–19.1)	93	7.9 (3.5–17.1)	26.6 (18.0–37.5)
Overall	134	9.4 (5.1–16.7)	7.6 (3.5–15.9)	138	10.7 (6.0–18.2)	31.9 (23.9–41.1)
Total in community						
65–74 years	603	7.0 (5.0–9.6)	4.4 (2.7–7.2)	3794	6.6 (5.8–7.4)	9.7 (8.8–10.7)
75–84 years	484	8.5 (5.7–12.5)	3.5 (1.8–6.7)	2838	6.7 (5.9–7.7)	10.8 (9.6–12.0)
≥85 years	182	10.2 (5.0–19.6)	2.0 (0.8–5.0)	899	6.0 (4.6–7.8)	9.6 (7.8–11.7)
Overall	1269	8.0 (6.2–10.1)	3.8 (2.6–5.5)	7531	6.5 (6.0–7.1)	10.1 (9.4–10.8)
Total in care homes						
65–74 years	26	17.7 (6.6–39.6)	7.5 (1.8–25.8)	18	11.3 (2.7–37.1)	30.3 (11.0–60.3)
75–84 years	69	11.7 (5.2–24.3)	8.5 (3.1–21.0)	62	16.2 (7.9–30.2)	37.0 (25.1–50.7)
≥85 years	88	7.2 (3.3–15.0)	6.6 (2.2–17.5)	112	6.7 (2.9–14.6)	25.5 (17.7–35.2)
Overall	183	10.0 (6.1–16.1)	7.4 (3.8–13.8)	192	9.8 (5.9–15.8)	29.2 (22.6–36.7)
By gender, standardised to 2011 age structure						
Men		5.9 (3.9–8.8)	3.1 (1.6–5.9)		4.5 (3.8–5.3)	6.4 (5.9–7.3)
Women		9.1 (6.9–11.9)	4.9 (3.1–7.6)		8.5 (7.7–9.5)	13.8 (12.7–15.0)
Total		7.9 (6.3–9.9)	4.2 (2.9–6.1)		6.8 (6.3–7.5)	10.7 (10.0–11.5)

CFAS, Cognitive Function and Ageing Studies.

a. Residential status missing for five individuals in CFAS I.

Within the three centres, individuals living in Newcastle were more likely to have case-level depression in CFAS I but less likely to be depressed in CFAS II (risk ratio Newcastle versus Cambridgeshire CFAS I: 3.21, 95% CI 1.56–6.59, *P* = 0.002; CFAS II 0.74, 95% CI 0.58–0.94, *P* = 0.027; test for interaction, *P* = 0.0001) (Table 2). Case-level depression was associated with living in a more deprived area in both studies. In CFAS I, the risk of depression was raised in all quartiles compared with the least deprived quartile (*P* = 0.018). In CFAS II, higher risk was observed in the most deprived quartile only (*P* = 0.0002).

**Table 2:** Adjusted^a^ risk ratios for sociodemographic factors and depression, CFAS I and CFAS II

	CFAS I risk ratio (95% CI)	*P-*value	CFAS II risk ratio (95% CI)	*P-*value	Test for interaction (CFAS I and CFAS II) *P-*value
Gender					
Male	Ref	0.40	Ref	<0.0001	0.16
Female	1.25 (0.74–2.11)		1.87 (1.55–2.26)		
Age group					
65–74 years	Ref	0.78	Ref	0.23	0.44
75–84 years	1.06 (0.64–1.76)		1.02 (0.85–1.26)		
≥85 years	1.30 (0.62–2.72)		0.79 (0.59–1.06)		
Residence					
Community	Ref	0.74	Ref	0.12	0.47
Care home	1.12 (0.59–2.13)		1.51 (0.89–2.57)		
Centre					
Cambridgeshire	Ref	0.0022	Ref	0.027	0.0001
Newcastle	3.21 (1.56–6.59)		0.74 (0.58–0.94)		
Nottingham	1.80 (0.84–3.84)		0.94 (0.76–1.17)		
Townsend deprivation index (quartiles)					
Q1 (least deprived)	Ref	0.018	Ref	0.0002	0.0096
Q2	2.18 (1.06–4.49)		0.89 (0.69–1.13)		
Q3	3.07 (1.51–6.25)		1.04 (0.82–1.33)		
Q4 (most deprived)	2.27 (1.08–4.75)		1.56 (1.22–2.00)		

CFAS, Cognitive Function and Ageing Studies; Ref, reference value; Q, quartile.

a. Risk ratios adjusted for all other covariates.

The proportion of older people receiving antidepressant medication in CFAS II was more than double that in CFAS I (adjusted risk ratio 2.79, 95% CI 1.96–3.97, *P* < 0.0001). Estimated prevalence of antidepressant use from the CFAS I cohort was 4.2% (95% CI 2.9–6.1%) and 10.7% (95% CI 10.0–11.5%) among CFAS II participants (Table 1). In CFAS II, after adjustment for other sociodemographics, women were more likely to be receiving antidepressant medication compared with men (risk ratio 2.05, 95% CI 1.76–2.39, *P* < 0.0001) (Table 3). There was no evidence that the relationship between gender and receiving antidepressant medication had changed since CFAS I (test for interaction, *P* = 0.78). As with the prevalence of case-level depression, there was no discernible age effect on antidepressant medication prescription. In care homes the use of antidepressants was nearly four times higher in CFAS II (29.2%, 95% CI 22.6–36.7%) than CFAS I (7.4%, 95% CI 3.8–13.8%). However, after adjustment for sociodemographic factors, the increased risk of receiving antidepressants for care home residence was similar to that of older people living in their own homes (CFAS I: risk ratio 3.07, 95% CI 1.47–6.42, *P* = 0.0029; CFAS II: risk ratio 2.76, 95% CI 2.12–3.61, *P* < 0.0001; test for interaction, *P* = 0.79), an indication of the change in the care home population.

**Table 3:** Adjusted^a^ risk ratios for sociodemographic factors and antidepressant treatment, CFAS I and CFAS II

	CFAS I risk ratio (95% CI)	*P*-value	CFAS II risk ratio (95% CI)	*P*-value	Test for interaction (CFAS I and CFAS II) *P*-value
Gender					
Male	Ref	0.15	Ref	<0.0001	0.78
Female	1.83 (0.81–4.11)		2.05 (1.76–2.39)		
Age group					
65–74 years	Ref	0.24	Ref	0.047	0.46
75–84 years	0.76 (0.34–1.69)		1.08 (0.93–1.25)		
≥85 years	0.47 (0.19–1.13)		0.82 (0.65–1.02)		
Residence					
Community	Ref	0.0029	Ref	<0.0001	0.79
Care home	3.07 (1.47–6.42)		2.76 (2.12–3.61)		
Centre					
Cambridgeshire	Ref	0.34	Ref	<0.0001	0.93
Newcastle	0.93 (0.37–2.36)		1.01 (0.84–1.20)		
Nottingham	0.58 (0.24–1.36)		0.68 (0.56–0.82)		
Townsend deprivation index (quartiles)					
Q1 (least deprived)	Ref	0.13	Ref	0.029	0.10
Q2	2.23 (0.87–5.75)		0.86 (0.71–1.05)		
Q3	0.85 (0.33–2.17)		0.95 (0.78–1.14)		
Q4 (most deprived)	1.60 (0.57–4.49)		1.19 (0.98–1.44)		

CFAS, Cognitive Function and Ageing Studies; Ref, reference value; Q, quartile.

a. Risk ratios adjusted for all other covariates.

**Fig. 1:**
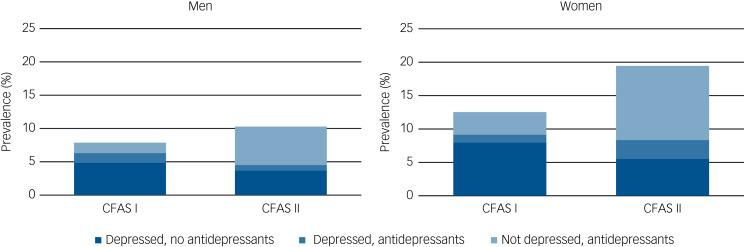
Prevalence of depression and antidepressant treatment by gender, CFAS I and CFAS II.

There was relatively little overlap between those who were receiving antidepressant medications and those reaching case-level diagnosis of depression at time of interview in either CFAS I or in CFAS II (Fig. 1, Supplementary Table 2). In CFAS I, 1.3% had study diagnostic-level depression and were receiving antidepressants, over 1 in 20 (6.8%) met study diagnostic level of depression but were not receiving antidepressants and a further 2.7% were receiving antidepressant medication but did not meet study diagnosis of depression. The equivalent proportions in CFAS II were 1.9%, 4.7% and 8.8%, respectively. In both cohorts, most people with case-level depression were not receiving antidepressant treatment and most of those receiving antidepressants did not have a study diagnosis of depression at time of interview.

CFAS, Cognitive Function and Ageing Studies.

## Discussion

From CFAS II we estimate that the prevalence of case-level depression is 6.8% in people aged ≥65 years. This was a relative but not statistically significant decrease of around 20% since CFAS I, conducted two decades earlier, after allowing for changes in age structure and other demographic differences. There was a threefold increase in antidepressant use over the same time period. Among CFAS I and CFAS II participants, only a minority of those with case-level depression were receiving antidepressant medications, and in both studies, most of those taking antidepressants did not have depression, with this proportion of the population increasing dramatically in CFAS II.

### Strengths and limitations

The analysis presented here is based on samples drawn from population-representative primary care registers that include residents of care homes, use diagnostic criteria held constant between two time points and are of sufficient scale to estimate prevalence. The approach that we used, direct interview by rigorously trained interviewers across sites, with standardised detailed questioning by validated methods, should ensure that our detection of depression is comparable across time. In drawing conclusions from our findings, the methodological limitations need to be considered. In spite of identical recruitment approaches, non-response in CFAS II was greater than in that achieved in CFAS I two decades earlier. However, the risk to biased estimates owing to lack of representativeness is limited by back-weighting of factors associated with non-response. Our measures of medication are based on what is reported as taken rather than what is prescribed, and it is not possible to determine the appropriateness or otherwise of antidepressant prescribing for study participants. Among older people, the level of non-adherence to medication^16^ presents challenges for studies based on prescription data.

The two-stage design (screening and assessment) of CFAS I limits the number of participants with diagnostic assessment, meaning the power to detect associations was greater at CFAS II. The analyses we report here are cross-sectional for each cohort, which limits our ability to comment on changes in duration of depressive or treatment episode. Both in the UK^17^ and elsewhere,^18^ psychological therapies are more widely used than at the time of CFAS I, but we did not ask participants about this directly and so cannot include non-medication treatment in our analysis.

### Findings in the context of the literature

CFAS I and CFAS II have allowed for direct comparison of changes in prevalence and treatment of depression among people aged ≥65 years across two decades. Prevalence estimates of depression are higher if samples are drawn from primary care attenders^19^ or where measurement is restricted to symptom scales.^20^ Our estimated prevalence of late-life depression from CFAS II is consistent with two reviews of epidemiological studies of comparable age groups. In the first, estimates of major depression according to ICD-10 or DSM-IV criteria ranged from 0.9 to 9.4%, with variation likely owing to the methodological and contextual differences of the reviewed studies.^21^ In the second, pooled prevalence from 13 studies was 7.2%.^3^

Prevalence of case-level depression decreased from 7.9% in CFAS I to 6.8% in CFAS II, but after adjustment this change was not statistically significant. Although other studies have looked at depression across time with the same methodology,^22–24^ their focus has been on younger age groups, making direct comparison difficult. A relatively short-term comparison from a repeat population interview survey between 1998 and 2004 of Australian adults of all ages found no change in prevalence of depression.^25^ Analyses of general practice attenders, where prevalence estimates will be higher than those from population studies, found that the incidence decreased from 22.5 to 14.0 per 1000 person-years between 1996 and 2006, and this decline was greater among those aged ≥65 years.^26^ In our analysis there was evidence of a change over time in the association between depression and living in a deprived area. That the risk appears to be confined to those in the most deprived quartile is perhaps indicative of changing social structures in England, with a decreasing proportion of the population being socioeconomically classified as ‘working class’.^27^

Taking antidepressant medication increased from 4.0 to 10.7% over the 20-year period. This is similar to the rise reported by others over a similar time period.^8–10^ Depression can be effectively treated by medication; therefore it is expected that there will be a substantial proportion of people taking medication, but not reporting the symptoms of depression. In CFAS I the number of those with untreated case-level depression was over twice that of those receiving antidepressant medication without a diagnosis. These proportions were reversed in CFAS II. There are a number of possible explanations for this apparent mismatch between prevalence and treatment, although the observational study design precludes any inference of a causal relationship. For those untreated, we cannot say if this is because of treatment not being offered, not being accepted, having been unsuccessful in the past or whether other, non-pharmacological treatments were being received. Participants who were not identified as depressed but were receiving antidepressant medications may have been treated successfully in the past and continue to take medications, perhaps preventing a rise in depression prevalence that might have been observed otherwise. Antidepressants may be more likely to be used to treat depression that does not reach the case-level threshold used in this study. Authors from other epidemiological studies have suggested that the need for treatment is poorly matched with provision.^28^

It is unclear whether observed increases in treatment are a reflection of overdiagnosis, better recognition and prescribing, or the prescribing of antidepressant medication for conditions other than depression. The comparisons made here were during a time of change in the way depression is detected and treated. In England, where most older people with depression are managed in primary care settings, policy shifts have been toward greater surveillance of those with chronic diseases. Depression directly affects 1 in 15 people aged ≥65 years, with its effects felt by families and friends. Over two decades, substantial increases in access to antidepressant medication do not appear to be associated with change in prevalence of late-life depression. The natural history of treated and untreated depression, particularly for older people, remains poorly understood.

## Supplementary material

For supplementary material accompanying this paper visit https://doi.org/10.1192/bjp.2019.193. click here to view supplementary material
