# Supplementary material for: Changing prevalence and treatment of depression among older people over two decades
Source: Br J Psychiatry. 2019 Oct 7;216(1):49–54. doi: 10.1192/bjp.2019.193 (PMC7557614; doi:10.1192/bjp.2019.193)
Supplement: Supplementary file 1 [file S0007125019001934sup001.docx]

| Supp Table 1: Characteristics of individuals participating in screen and assessment (CFAS I) and interview (CFAS II) | | | |
| --- | --- | --- | --- |
|  | CFAS 1 |  | CFAS II^a^ |
|  | Screening^a^  n (%) | Assessment^b^  n (%) | n (%) |
| Sex | | | |
| Men | 3,045 (39.2) | 531 (37.6) | 3,534 (43.9) |
| Women | 4,590 (60.8) | 926 (62.4) | 4,228 (56.1) |
| Age group | | | |
| 65-69 years | 1,981 (25.0) | 310 (23.0) | 1,939 (23.0) |
| 70-74 years | 1,776 (22.8) | 320 (21.5) | 1,873 (22.7) |
| 75-79 years | 1,725 (22.5) | 263 (23.5) | 1,624 (20.5) |
| 80-84 years | 1,308 (17.7) | 291 (19.5) | 1,278 (17.5) |
| 85-89 years | 615 (8.5) | 186 (9.4) | 737 (10.5) |
| +90 years | 230 (3.5) | 87 (3.1) | 311 (5.8) |
| Location | | | |
| Cambridgeshire | 2,601 (34.2) | 465 (37.5) | 2,558 (30.2) |
| Newcastle | 2,522 (32.9) | 499 (30.5) | 2,582 (34.5) |
| Nottingham | 2,512 (32.9) | 493 (32.0) | 2,622 (35.4) |
| Residential status | | | |
| Community | 7,282 (95.2) | 1,269 (94.8) | 7,565 (96.7) |
| Care homes | 346 (4.8) | 183 (5.2) | 197 (3.3) |

| Supp table 2: Depression and treatment with antidepressants by demographic factors (CFAS I & CFAS II) | | | | | | | | | |
| --- | --- | --- | --- | --- | --- | --- | --- | --- | --- |
|  | CFAS I | | | |  | CFAS II | | | |
|  | Depression  ADs^1^ | Depression  No ADs | No Depression  ADs | No Depression  No ADs |  | Depression  ADs | Depression  No ADs | No Depression  ADs | No Depression  No ADs |
| All | 26 (1.3) | 126 (6.8) | 47 (2.7) | 1258 (89.3) |  | 145 (1.9) | 356 (4.7) | 654 (8.8) | 6568 (84.6) |
| Male  Female | 9 (1.5)  17 (1.2) | 39 (4.8)  87 (8.0) | 10 (1.4)  37 (3.4) | 473 (92.3)  785 (87.5) |  | 30 (0.8)  115 (2.8) | 127 (3.7)  229 (5.5) | 199 (5.8)  455 (11.1) | 3169 (89.7)  3399 (80.6) |
| Age group  65-74 years  75-84 years  ≥85 years | 12 (1.3)  11 (1.3)  3 (0.9) | 55 (5.8)  51 (7.3)  20 (8.6) | 23 (3.1)  18 (2.4)  6 (2.1) | 540 (89.8)  474 (89.0)  244 (88.4) |  | 76 (2.0)  49 (1.8)  20 (2.0) | 167 (4.6)  147 (5.1)  42 (4.1) | 282 (7.8)  272 (9.5)  100 (9.6) | 3287 (85.6)  2432 (83.5)  849 (84.3) |
| Residence  Community  Care home | 20 (1.1)  6 (3.8) | 114 (6.8)  12 (6.2) | 42 (2.6)  5 (3.6) | 1093 (89.4)  160 (86.4) |  | 137 (1.8)  8 (5.2) | 349 (4.7)  7 (4.6) | 604 (8.3)  50 (24.0) | 6441 (85.2)  127 (66.2) |
| Centre  Cambridgeshire  Newcastle  Nottingham | 7 (0.6)  9 (1.5)  10 (1.8) | 25 (2.9)  65 (12.2)  36 (6.2) | 20 (4.2)  16 (2.3)  11 (1.2) | 413 (92.2)  409 (84.0)  436 (90.8) |  | 46 (1.8)  55 (2.2)  44 (1.8) | 119 (4.6)  97 (4.0)  140 (5.5) | 222 (8.9)  273 (11.1)  159 (6.4) | 2171 (84.6)  2119 (82.7)  2278 (86.4) |
| Townsend deprivation index  Q1  Q2  Q3  Q4 | 5 (0.7)  6 (1.0)  6 (1.3)  9 (2.5) | 16 (2.5)  23 (5.2)  42 (11.6)  40 (8.4) | 9 (2.5)  16 (5.6)  11 (1.1)  11 (1.7) | 259 (94.4)  286 (88.2)  312 (85.9)  336 (87.4) |  | 41 (1.8)  28 (1.3)  39 (2.2)  37 (2.5) | 95 (4.3)  88 (4.2)  76 (4.1)  97 (6.2) | 182 (8.3)  155 (8.1)  148 (8.1)  169 (10.6) | 1946 (85.6)  1787 (86.4)  1563 (85.6)  1272 (80.8) |
